# External validation of a claims-based algorithm for classifying kidney-cancer surgeries

**DOI:** 10.1186/1472-6963-9-92

**Published:** 2009-06-06

**Authors:** David C Miller, Christopher S Saigal, Joan L Warren, Meryl Leventhal, Dennis Deapen, Mousumi Banerjee, Julie Lai, Jan Hanley, Mark S Litwin

**Affiliations:** 1 Department of Urology, University of Michigan, Ann Arbor, MI, 48109, USA; 2 Center for Clinical Management Research, VA Ann Arbor Healthcare System, Ann Arbor, MI, USA; 3 Department of Urology, University of California, Los Angeles, Los Angeles, CA, USA; 4 Jonsson Comprehensive Cancer Center, University of California, Los Angeles, Los Angeles, CA, USA; 5 RAND Health, RAND Corporation, Santa Monica, CA, USA; 6 Applied Research Program, National Cancer Institute, Bethesda Md, USA; 7 Cancer Surveillance Program, University of Southern California, Los Angeles, CA, USA; 8 Department of Biostatistics, University of Michigan School of Public Health, Ann Arbor, MI, USA; 9 Comprehensive Cancer Center, University of Michigan, Ann Arbor, MI, USA; 10 Department of Health Services, School of Public Health, University of California, Los Angeles, CA, USA

## Abstract

**Background:**

Unlike other malignancies, there is no literature supporting the accuracy of medical claims data for identifying surgical treatments among patients with kidney cancer. We sought to validate externally a previously published Medicare-claims-based algorithm for classifying surgical treatments among patients with early-stage kidney cancer. To achieve this aim, we compared procedure assignments based on Medicare claims with the type of surgery specified in SEER registry data and clinical operative reports.

**Methods:**

Using linked SEER-Medicare data, we calculated the agreement between Medicare claims and SEER data for identification of cancer-directed surgery among 6,515 patients diagnosed with early-stage kidney cancer. Next, for a subset of 120 cases, we determined the agreement between the claims algorithm and the medical record. Finally, using the medical record as the reference-standard, we calculated the sensitivity, specificity, and positive and negative predictive values of the claims algorithm.

**Results:**

Among 6,515 cases, Medicare claims and SEER data identified 5,483 (84.1%) and 5,774 (88.6%) patients, respectively, who underwent cancer-directed surgery (observed agreement = 93%, κ = 0.69, 95% CI 0.66 – 0.71). The two data sources demonstrated 97% agreement for classification of partial versus radical nephrectomy (κ = 0.83, 95% CI 0.81 – 0.86). We observed 97% agreement between the claims algorithm and clinical operative reports; the positive predictive value of the claims algorithm exceeded 90% for identification of both partial nephrectomy and laparoscopic surgery.

**Conclusion:**

Medicare claims represent an accurate data source for ascertainment of population-based patterns of surgical care among patients with early-stage kidney cancer.

## Background

The introduction of partial nephrectomy (also called kidney-sparing surgery) and laparoscopy (also called minimally-invasive surgery) changed the paradigm for surgical management of patients with early-stage kidney cancer in two important ways.[1] First, partial nephrectomy – which achieves equal rates of cure while better preserving long-term renal function – replaced radical nephrectomy (complete excision of the tumor-bearing kidney) as the favored treatment for patients with small renal tumors.[2,3] Second, for many patients undergoing radical nephrectomy, laparoscopy became the recommended surgical approach because it affords easier convalescence than conventional incisional surgery without compromising long-term cancer control.[4]

Despite their potential benefits to patients, surgeon adoption of partial nephrectomy and laparoscopy has been gradual and assymetric, [5-7] suggesting an opportunity to achieve population-level improvements in the quality of surgical care for patients with kidney cancer. In an effort to understand this protracted dissemination, we recently used linked data from the National Cancer Institute's Surveillance, Epidemiology and End Results (SEER) program and the Centers for Medicare and Medicaid Services (Medicare) to estimate the magnitude of surgeon- versus patient-attributable variance in the use of partial nephrectomy and laparoscopy.[7] For that study, we implemented a Medicare-claims-based algorithm to identify and classify the procedure received by each subject treated surgically for early-stage kidney cancer. The claims-based algorithm included decision-rules to resolve discrepancies between hospital and physician procedure claims (e.g., a hospital claim for partial nephrectomy coupled with a physician claim for radical nephrectomy).[7]

Unlike prostate, lung, and breast cancer, [8,9]however, no published literature supports the accuracy of claims data for identifying surgical treatments among patients with kidney cancer. Moreover, the initial absence of specific administrative codes for laparoscopic kidney cancer surgeries presented a special challenge for claims-based assessments of evolving surgical practice patterns. The primary goal of this study, therefore, was to validate our Medicare-claims-based algorithm for classifying kidney cancer surgeries by comparing procedure assignments based on Medicare claims with the type of surgery specified in SEER registry data and actual clinical operative reports. Our findings will, in turn, further clarify the integrity of claims data as a tool for studying patterns of surgical care among patients with kidney cancer.

## Methods

### Identification of study cohort and development of claims algorithm

We sought to validate a Medicare-claims-based algorithm for surgical procedure assignment employed in a previously-published study (referred to as the parent study) that used linked SEER-Medicare data to study the utilization of partial nephrectomy and laparoscopic radical nephrectomy among Medicare beneficiaries diagnosed with kidney cancer from 1997 through 2003.[7]SEER is a population-based cancer registry that collects data regarding incidence, treatment, and mortality. The demographic composition, cancer incidence, and mortality trends in the SEER registries are representative of the entire United States population.[10]The Medicare program provides primary health insurance for 97% of the United States population ages ≥ 65 years. Successful linkage with Medicare claims is achieved for > 90% of Medicare patients whose cancer-specific data are tracked by SEER.[11]

From the SEER data included with linked SEER-Medicare files, we identified 6,515 eligible subjects with a new diagnosis of localized/regional, non-urothelial kidney cancer (see Additional File 1). For each case in this preliminary cohort, we searched Medicare inpatient (International Classification of Diseases, 9th revision, Clinical Modification (ICD-9) codes) and physician (American Medical Association Current Procedural Terminology (CPT) and ICD-9 codes) claims for kidney cancer-specific diagnosis and surgical procedure codes (see Additional File 2). Using this approach, we identified an analytic cohort comprising 5,483 patients (84.2% of the preliminary cohort) with Medicare claims specifying the occurrence of cancer-directed surgical therapy (see Additional File 1).[7]

We then applied a claims-based algorithm (defined *a priori*) to classify the specific type of surgical therapy received by each patient (see Additional File 3). The algorithm included explicit rules for procedure assignment for cases with multiple surgical claims and/or discrepancies between the inpatient and physician claims (e.g., a hospital claim for partial nephrectomy coupled with a physician claim for radical nephrectomy). We used both direct (CPT) and indirect (CPT and ICD-9) codes to identify cases performed laparoscopically (see Additional Files 2 and 3). As part of this algorithm, we also ascribed a laparoscopic approach to patients whose length of hospital stay was ≤ 2 days following radical or partial nephrectomy. By employing this algorithm, we assigned each case to one of four mutually exclusive surgical categories: (1) open radical nephrectomy (ORN); (2) open partial nephrectomy (OPN); (3) laparoscopic radical nephrectomy (LRN); (4) laparoscopic partial nephrectomy (LPN).[7] Because we anticipated small numbers during the specified study interval, we included patients undergoing kidney-sparing, probe-ablative (i.e., cryo or radiofrequency) therapies in the partial nephrectomy cohorts.

### Validation of claims algorithm

After obtaining Institutional Review Board approvals, we undertook a two-step approach to assessing the validity of our claims-based algorithm for kidney cancer surgical procedure assignment. First, we assessed the level of concordance between Medicare claims and SEER data for identification of cancer-directed surgery (i.e., ablative surgery, partial nephrectomy, or radical nephrectomy) among 6,515 patients in the preliminary cohort from our parent study (see Additional File 1).[8,9] We did this by calculating both the observed agreement between the two data sources (i.e., the number of cases in agreement divided by the total number of cases) and the corresponding kappa statistic, which quantifies the degree of agreement, adjusting for chance. We used the following scale for assessing strength of agreement based on kappa statistics: κ = 0.81–1.00 almost perfect agreement; κ = 0.61–0.80 substantial agreement; κ = 0.41–0.60 moderate agreement; κ = 0.21–0.40 fair agreement.[12] For cases where cancer-directed surgery was specified in both Medicare claims and SEER data, we used the same approach to assess the level of agreement between data sources for identification of partial versus radical nephrectomies. Using SEER data as the reference-standard, we additionally calculated the sensitivity, specificity, and positive (PPV) and negative (NPV) predictive values for the claims algorithm (both for the identification of any cancer-directed surgery and for the identification of partial nephrectomies). All statistical testing was completed using computerized software (SAS v9.1, SAS Institute, Cary, NC).

For the second step, we collaborated with the SEER registry for Los Angeles County, the Cancer Surveillance Program (CSP) at the University of Southern California, to carry out a medical-records-based validation of our claims algorithm. In order to retrieve medical records, we provided the CSP with a de-identified list of patient codes for Los Angeles cases included in the analytic cohort for the parent study (n = 549). These 549 cases from the parent study matched with 605 records in the CSP (for patients treated at more than one hospital a report is collected from each). After the initial match – and before sampling cases – CSP staff excluded 14 LA County residents who were diagnosed and/or treated in facilities outside of LA County, 15 cases from facilities that are now closed, and 8 cases whose CSP records did not include a code for kidney cancer surgery. Anticipating that future studies in this area will employ currently-available procedure codes, we decided *a priori *to stratify the remaining eligible cases according to treatment year (2000–2003 vs 1997–1999); CSP staff then sampled 5 cases from 2000–2003 for every 1 case from 1997–1999. CSP staff requested information for 141 cases before successfully retrieving records for the 120 cases (22% of Los Angeles cases, 2% of the total sample from the parent study) comprising our final validation sample (i.e., medical records were not available for 21 patients). Detailed patient characteristics for each of the previously described cohorts and sub-cohorts are presented in Additional File 4.

Upon receipt of records from treating facilities, CSP chart abstractors identified and photocopied the kidney cancer surgery operative report (or the relevant hospital discharge summary for one case). Next, they removed all patient and/or provider-level identifiers, replaced these with the corresponding unique identifiers from the parent study, and sent the de-identified, photocopied medical records back to UCLA for review and data abstraction. One study author (DCM) reviewed the medical records (operative note for 119 cases, discharge summary for 1 case) to determine the surgical procedure received by each patient (ORN, OPN, LRN, LPN).

In analyses for the subset of cases with medical records data, we considered the procedure specified in the operative note to be the reference-standard. We first calculated the overall concordance or observed agreement rate between the claims algorithm and the medical record (i.e., the number of cases where procedure assignment was the same for the claims algorithm and the operative report divided by the total number of cases in the validation sample). We performed separate calculations for overall procedure assignments (i.e., ORN vs OPN vs LRN vs LPN), partial versus radical nephrectomy, and open versus laparoscopic surgical approach. Finally, we calculated the sensitivity, specificity, PPV and NPV of the claims algorithm for identification of open surgery versus laparoscopy, and partial versus radical nephrectomy.

## Results

Among 6,515 Medicare beneficiaries with a new diagnosis of localized/regional, non-urothelial kidney cancer, Medicare claims and SEER data identified 5,483 (84.1%) and 5,774 (88.6%) patients, respectively, who underwent cancer-directed surgery. For cases with both hospital and physician claims specifying the occurrence of cancer-directed surgery, we observed 98% agreement (κ = 0.88, 95% CI 0.86 – 0.90) for classification of partial versus radical nephrectomy, and 94% agreement (κ = 0.45, 95% CI 0.40 – 0.50) for classification of laparoscopic versus open surgery.

As presented in Table 1, agreement between the two data sources exceeded 90% (κ = 0.69, 95% CI 0.66 – 0.71). The sensitivity of the claims algorithm for identifying the occurrence of kidney cancer-directed surgery was 93.3% (95% CI 92.7%–94.0%), with a PPV of 98.3% (95% CI 97.9%–98.6%) (Table 1). Among the 5,389 cases where both the claims algorithm and SEER data specified the occurrence of cancer-directed surgery, we observed 97% agreement between data sources for classification of partial versus radical nephrectomy (κ = 0.83, 95% CI 0.81 – 0.86) (Table 2).

**Table 1:** Agreement between claims algorithm and SEER data for receipt of cancer-directed surgery among patients with kidney cancer

		**RECEIPT OF SURGERY BASED ON MEDICARE CLAIMS**		
		**No**	**Yes**	**Total**
**RECEIPT OF SURGERY BASED ON SEER CANCER-DIRECTED SURGERY VARIABLE**	**No**	647	94	741
	
	**Yes**	385	5389	5774
	
	**Total**	1032	5483	6515
				
**Observed agreement = 92.6%**				
**Kappa = 0.69 (0.66–0.71)**				
**Sensitivity^† ^= 93.3% (92.7%–94.0%)**				
**Specificity^† ^= 87.3% (84.7%–89.6%)**				
**Positive Predictive Value^† ^= 98.3% (97.9%–98.6%)**				
**Negative Predictive Value^† ^= 62.7% (59.7%–65.7%)**				

† Calculations based on SEER cancer-directed surgery variable as the reference-standard

**Table 2:** Agreement between claims algorithm and SEER data for identification of partial versus radical nephrectomy

		**PROCEDURE BASED ON MEDICARE CLAIMS**		
		**Partial Nephrectomy**	**Radical Nephrectomy**	**Total**
**PROCEDURE BASED ON SEER CANCER-DIRECTED SURGERY VARIABLE**	**Partial Nephrectomy**	495	86	581
	
	**Radical Nephrectomy**	88	4720	4808
	
	**Total**	583	4806	5389
**Observed agreement = 96.8%**				
**Kappa = 0.83 (0.81–0.86)**				
**Sensitivity^† ^= 85.2% (82.0%–88.0%)**				
**Specificity^† ^= 98.2% (97.7%–98.5%)**				
**Positive Predictive Value^† ^= 84.9% (81.7%–87.7%)**				
**Negative Predictive Value^† ^= 98.2% (98.0%–98.6%)**				

† Calculations are for identification of partial nephrectomies based on SEER cancer-directed surgery variable as the reference-standard

Among the 120 subjects whose medical records were reviewed, 100 (83%) and 20 (17%) underwent surgery in 2000–2003 and 1997–1999, respectively. The claims-based algorithm correctly classified the specific surgical procedure for 116 of 120 cases (observed agreement = 96.7%) (Table 3). The four misclassified cases include the following: 1) one case classified as an open radical nephrectomy was actually a laparoscopic radical nephrectomy; 2) one case classified as a laparoscopic radical nephrectomy was actually an open radical nephrectomy (although it had started as a laparoscopic case before the surgeon converted to conventional open surgery); 3) one case classified as a laparoscopic radical nephrectomy was actually a laparoscopic partial nephrectomy; and 4) one case classified as a laparoscopic partial nephrectomy was actually a percutaneous ablative procedure (for analytic purposes we assigned this case to the open partial nephrectomy group since the misclassification was based only on surgical approach). For the single case where we reviewed only the hospital discharge summary, both the claims algorithm and the medical record specified an open partial nephrectomy.

**Table 3:** Agreement between claims algorithm and operative reports for specific kidney cancer surgical procedures

		**PROCEDURE BASED ON OPERATIVE REPORT**				
		**LPN**	**OPN**	**LRN**	**ORN**	**Total**
**PROCEDURE BASED ON MEDICARE CLAIMS**	**LPN**	2	1*	0	0	3
	
	**OPN**	0	14	0	0	14
	
	**LRN**	1	0	8	1	10
	
	**ORN**	0	0	1	92	93
	
	**Total**	3	15	9	93	120
	
**Observed agreement = 96.7%**						

*This case actually represents a percutaneous ablation of a kidney tumor; however, for analytic purposes, we classified it as an open partial nephrectomy since the misclassification was based on surgical approach

Tables 4 and 5 compare case classifications based on the claims algorithm and operative reports for partial vs radical nephrectomy and for laparoscopic vs open surgery, respectively. The PPV of the claims algorithm exceeded 90% for identification of both partial nephrectomy (Table 4) and laparoscopic (Table 5) surgeries.

**Table 4:** Agreement between claims algorithm and operative reports for partial versus radical nephrectomy

		**PROCEDURE BASED ON OPERATIVE REPORT**		
		**Partial Nephrectomy**	**Radical Nephrectomy**	**Total**
**PROCEDURE BASED ON MEDICARE CLAIMS**	**Partial Nephrectomy**	16	1	17
	
	**Radical Nephrectomy**	1	102	103
	
	**Total**	17	103	120
**Observed agreement = 98.3%**				
**Sensitivity^† ^= 94.1% (71.3%–99.9%)**				
**Specificity^† ^= 99.0% (94.7%–99.9%)**				
**Positive Predictive Value^† ^= 94.1% (71.3%–99.9%)**				
**Negative Predictive Value^† ^= 99.0% (94.7%–99.9%)**				

† Calculations are for identification of partial nephrectomies based on the operative report (medical record) as the reference-standard

**Table 5:** Agreement between claims algorithm and operative reports for laparoscopic versus open kidney cancer surgeries

		**PROCEDURE BASED ON OPERATIVE REPORT**		
		**Laparoscopy**	**Open Surgery**	**Total**
**PROCEDURE BASED ON MEDICARE CLAIMS**	**Laparoscopy**	11	1	12
	
	Open Surgery*	2	106	108
	
	**Total**	13	107	120
**Observed agreement = 97.5%**				
**Sensitivity^† ^= 84.6% (54.6%–98.1%)**				
**Specificity^† ^= 99.0% (94.9%–99.9%)**				
**Positive Predictive Value^† ^= 91.7% (61.5%–99.8%)**				
**Negative Predictive Value^† ^= 98.1% (93.5%–99.8%)**				

† Calculations are for identification of laparoscopic cases based on the operative report (medical record) as the reference-standard

* includes one percutaneous ablative procedure

## Discussion

Administrative and tumor registry data are useful tools for researchers interested in the delivery and outcomes of cancer care. For instance, linked SEER-Medicare data can be used to study population-based trends in cancer screening, diagnosis, treatment, and survivorship among Medicare beneficiaries in the United States.[11] In this study, we evaluated the validity of Medicare claims for determining the type of surgical therapy received by patients with early-stage kidney cancer. We report three principal findings. First, we observed substantial agreement (κ = 0.69) between Medicare claims and SEER registry data for the identification of cancer-directed surgery among patients with early-stage kidney cancer. Second, our claims-based algorithm assigned the correct surgical procedure (ORN, OPN, LRN, or LPN) for 97% of the subset of cases for whom we performed medical record review. Third, our claims algorithm demonstrated a high positive predictive value for identification of partial nephrectomy (PPV 94%) and laparoscopic kidney cancer surgeries (PPV 92%). The consequent inference is that Medicare claims represent an accurate data source for ascertainment of population-based patterns of surgical care among patients with early-stage kidney cancer.

Our findings augment existing literature that describes the fidelity of claims data for evaluating practice patterns in surgical oncology.[8,13] Prior work has demonstrated good agreement between SEER and Medicare claims for identification of patients undergoing major surgical resections for breast, colorectal, endometrial, lung, pancreatic or prostate cancer.[8] Moreover, medical record reviews have confirmed the high sensitivity and positive predictive value of Medicare claims for detection of patients undergoing inpatient colectomy, mastectomy, or hysterectomy.[13]Data from the current study suggest that Medicare claims can also be used to classify accurately patients undergoing partial or radical nephrectomy for kidney cancer (by either an open or laparoscopic approach). The ability to distinguish laparoscopic from open surgical cases is particularly relevant since these data are not routinely available from the SEER registries. Accordingly, medical claims (available from Medicare and other payers) represent a valid and reliable source of data for monitoring – at a national level – the impact of ongoing initiatives that seek to increase surgeons' use of partial nephrectomy and laparoscopy.

Our study has several limitations. First, the small sample size for the medical record validation may raise concerns about the generalizability of our findings for the greater population of Medicare beneficiaries with kidney cancer. Second, because CSP abstractors were unable to locate medical records for some patients, it is possible that we have overestimated the concordance between the claims algorithm and surgeon operative notes. Third, energy ablative therapies (i.e., cryosurgery and radiofrequency ablation) are now used more frequently as alternatives to partial nephrectomy. Because we anticipated (and observed) very small numbers of these cases in the parent study, we included ablative therapies with the partial nephrectomies. In the future, updated claims algorithms may be needed to separately identify patients treated with ablative therapies. Finally, the generalizability of our results may be limited by a sample that includes only patients ≥ 66 years of age at the time of diagnosis. We do not believe, however, that patterns of treatment are systematically different for 65-year-olds versus older Medicare beneficiaries ages 66 and older.

## Conclusion

Despite these limitations, this study confirms a high level of agreement between Medicare claims and clinical operative reports for the identification of specific kidney cancer surgeries. Claims data, therefore, may be considered a valid substrate for studying patterns of surgical care among patients with kidney cancer.

## List of abbreviations

ORN: Open Radical Nephrectomy; OPN: Open Partial Nephrectomy; LRN: Laparoscopic Radical Nephrectomy; LPN: Laparoscopic Partial Nephrectomy; CSP: Cancer Surveillance Program (Los Angeles); SEER: Surveillance, Epidemiology, and End Results; PPV: Positive Predictive Value; NPV: Negative Predictive Value; LA: Los Angeles.

## Competing interests

The authors declare that they have no competing interests.

## Authors' contributions

DM conceived of the study, participated in its design, coordination, execution and analyses, and drafted the manuscript. CS conceived of the study, participated in its design, coordination and analyses, and helped to draft the manuscript. JW participated in the study design and analyses, and helped to draft the manuscript. ML participated in the study design and coordinated ascertainment of cases from the LA Tumor Registry. DD participated in the study design and coordination, supervised ascertainment of cases from the LA Tumor Registry, and helped to draft the manuscript. MB participated in the study design and statistical analyses and helped to draft the manuscript. JL and JH performed statistical analyses for the study. ML conceived of the study, participated in its design, coordination and analyses, and drafted the manuscript. All authors read and approved the final manuscript. 

## Pre-publication history

The pre-publication history for this paper can be accessed here:



## Supplementary Material

Additional file 1 **Cohort Selection**. Flow diagram describing selection of cohort for parent study. Click here for file

Additional file 2 **Identification of cases treated surgically for kidney cancer**. This file contains additional details (including tables with specific CPT and ICD-9 procedural codes) regarding our method for identifying Medicare beneficiaries who underwent surgical treatment for early-stage kidney cancer. Click here for file

Additional file 3 **Description of algorithms for surgical procedure assignment**. The series of tables in Additional File 3 illustrate our algorithm for surgical procedure assignment. This algorithm included methods to resolve procedure assignment for cases with more than one relevant physician procedural claim (Additional File 3, Table A), more than one relevant inpatient (i.e., hospital) procedural claim (Additional File 3, Table B), and/or a discrepancy between inpatient and physician claims (Additional File 3, Table C). Click here for file

Additional file 4 **Distribution of patient characteristics**. The series of tables in Additional File 4 summarize the distribution of patient characteristics for each of the patient cohorts and sub-cohorts specified in the manuscript. Click here for file

## References

[B1] Novick AC (2004). Laparoscopic and partial nephrectomy. Clinical Cancer Research.

[B2] Fergany AF, Hafez KS, Novick AC (2000). Long-term results of nephron sparing surgery for localized renal cell carcinoma: 10-year followup. J Urol.

[B3] Huang WC, Levey AS, Serio AM, Snyder M, Vickers AJ, Raj GV (2006). Chronic kidney disease after nephrectomy in patients with renal cortical tumours: a retrospective cohort study. Lancet Oncol.

[B4] Dunn MD, Portis AJ, Shalhav AL, Elbahnasy AM, Heidorn C, McDougall EM (2000). Laparoscopic versus open radical nephrectomy: a 9-year experience. J Urol.

[B5] Miller DC, Hollingsworth JM, Hafez KS, Daignault S, Hollenbeck BK (2006). Partial nephrectomy for small renal masses: an emerging quality of care concern?. J Urol.

[B6] Miller DC, Taub DA, Dunn RL, Wei JT, Hollenbeck BK (2006). Laparoscopy for renal cell carcinoma: diffusion versus regionalization?. J Urol.

[B7] Miller DC, Saigal CS, Banerjee M, Hanley J, Litwin MS (2008). Diffusion of surgical innovation among patients with kidney cancer. Cancer.

[B8] Cooper GS, Virnig B, Klabunde CN, Schussler N, Freeman J, Warren JL (2002). Use of SEER-Medicare data for measuring cancer surgery. Med Care.

[B9] Cooper GS, Yuan Z, Stange KC, Dennis LK, Amini SB, Rimm AA (2000). Agreement of Medicare claims and tumor registry data for assessment of cancer-related treatment. Med Care.

[B10] Hankey BF, Ries LA, Edwards BK (1999). The surveillance, epidemiology, and end results program: a national resource. Cancer Epidemiology, Biomarkers & Prevention.

[B11] Warren JL, Klabunde CN, Schrag D, Bach PB, Riley GF (2002). Overview of the SEER-Medicare data: content, research applications, and generalizability to the United States elderly population. Med Care.

[B12] Fleiss JL (1981). Statistical Methods for Rates and Proportions.

[B13] McClish DK, Penberthy L, Whittemore M, Newschaffer C, Woolard D, Desch CE (1997). Ability of Medicare claims data and cancer registries to identify cancer cases and treatment. Am J Epidemiol.

