# Supplementary material for: External validation of a claims-based algorithm for classifying kidney-cancer surgeries
Source: BMC Health Serv Res. 2009 Jun 6;9:92. doi: 10.1186/1472-6963-9-92 (PMC2698842; doi:10.1186/1472-6963-9-92)
Supplement: Additional file 1 — Cohort Selection. Flow diagram describing selection of cohort for parent study. [file 1472-6963-9-92-S1.doc]

**6,515** Medicare beneficiaries with a new diagnosis of localized/regional, non-urothelial kidney cancer

**“preliminary cohort” for parent study**

**15,741** patients ***in SEER*** with localized/regional kidney cancer

(*International Classification of Diseases*–Oncology 3 code C64.9)

Excluded (*n* =9,226)

• cases with a kidney-cancer diagnosis based solely on autopsy

findings or death-certificate documentation 109

• cases with histology codes specifying a diagnosis of urothelial

carcinoma of the renal pelvis and/or collecting system565

• cases enrolled in a Medicare Health Maintenance

Organization (medical claims are not submitted to the CMS)2,589

• cases < 66 or > 90 years of age at diagnosis (to ensure

12-month Medicare eligibility window for ascertaining

comorbidity)5,316

• cases lacking continuous Medicare enrollment

(both Parts A and B) for at least 12 months before and

6 months after kidney-cancer diagnosis647

**Additional File 1. Cohort Selection**

Excluded (*n*=1,032)

• cases with either no surgical claims (*n*=1,026) or 2 separate admissions for kidney cancer surgery (*n*=3 patients, or 6 cases)

• ***specific Medicare claims used to identify patients undergoing kidney cancer surgery are summarized in Additional File 2***

**5,483** patients (84.2% of the “preliminary cohort”) with Medicare claims specifying surgical treatment for early-stage kidney cancer

**“analytic cohort” for parent study**
