# Supplementary material for: External validation of a claims-based algorithm for classifying kidney-cancer surgeries
Source: BMC Health Serv Res. 2009 Jun 6;9:92. doi: 10.1186/1472-6963-9-92 (PMC2698842; doi:10.1186/1472-6963-9-92)
Supplement: Additional file 2 — Identification of cases treated surgically for kidney cancer. This file contains additional details (including tables with specific CPT and ICD-9 procedural codes) regarding our method for identifying Medicare beneficiaries who underwent surgical treatment for early-stage kidney cancer. [file 1472-6963-9-92-S2.doc]

**Additional File 2. Identification of cases treated surgically for early-stage kidney cancer**

For each case in our preliminary cohort of 6,515 Medicare beneficiaries diagnosed between 1997 and 2002 with localized/regional, non-urothelial kidney cancer, we searched both inpatient (Medicare Provider Analysis and Review [MEDPAR] file, based on International Classification of Diseases, 9th revision, Clinical Modification (ICD-9) codes) and physician claims (Carrier Claims file, based on American Medical Association Current Procedural Terminology (CPT) and ICD-9 codes) for kidney cancer–specific diagnosis and procedural codes (see Table below).

Of these cases, we excluded 1,026 who lacked claims denoting surgical treatment for kidney cancer. We also excluded 3 patients (6 cases) whose claims suggested the presence of bilateral tumors at diagnosis. We retained for analysis 4,965 cases who met the following criteria: (1) an inpatient claim (i.e., hospital admission) for kidney cancer surgery with an associated kidney cancer diagnosis; (2) a physician claim for kidney cancer surgery with an associated kidney cancer diagnosis; and (3) date correspondence (± 30 days) between the hospital and physician claims. We also retained 83 cases that met a secondary set of conditions for inclusion: (1) an inpatient claim that includes either a diagnostic or surgical code for kidney cancer (but not both); (2) a physician claim for kidney cancer surgery with an associated kidney cancer diagnosis; and (3) a date for the physician claim that fell between the admission and discharge dates for the corresponding hospital stay. Finally, we retained for analysis 435 additional cases with an inpatient claim for kidney cancer surgery and a corresponding kidney cancer diagnosis codes, but no matching physician claim. This process yielded a final analytic cohort of 5,483 cases (84.2% of the preliminary cohort).

**Additional File** 2, Table. Diagnosis and procedural codes used to identify patients treated surgically for early-stage kidney cancer

| **ICD-9 Diagnosis Codes** | **Description** |
| --- | --- |
| 189, 1890, 18900, 18901 | Malignant neoplasm of the kidney except renal pelvis |
| 1898, 1899 | Malignant neoplasm of other or unspecified site, urinary tract origin |
| 1580 | Malignant neoplasm of the retroperitoneum |
| 1715 | Malignant neoplasm of the connective and soft tissue of the abdomen |
| 1940 | Malignant neoplasm of the adrenal gland |
| 1952 | Malignant neoplasm of the abdomen |
| 1976, 1980, 1981, 19889, 1991 | Second malignant neoplasm of the retroperitoneum, kidney, other urinary organ, other specified site, unspecified site |
| 2118, 2230, 2231, 22389, 2239 | Benign neoplasm of the retroperitoneum, kidney and other urinary organ, kidney except pelvis, other urinary organ, unspecified urinary organ |
| 2339 | Carcinoma in-situ involving urinary organs |
| 2354, 2372, 2381, 2388 | Neoplasm of uncertain behavior of the retroperitoneum, adrenal gland, connective/soft tissue, other specified site |
| 2369, 23690, 23691, 23699, 2395, 2399 | Neoplasm of uncertain behavior of the kidney and ureter, other specified urinary organ, unspecified urinary organ |
| 4532, 4533 | Embolism and thrombosis of vena cava, renal vein |
| 593, 5931, 5932, 5938, 59389, 5939, 59390 | Other disorders of the kidney and ureter |
| 5997 | Hematuria |
| 75310, 75311 | Cystic kidney disease |
| 7890, 78900, 78901, 78902, 78903, 78904, 78905, 78906, 78907, 78909 | Abdominal pain |
| 78930, 78931, 78932, 78934, 78937, 78939 | Abdominal/pelvic mass or swelling |
| 7935, 7939 | Abnormal radiographic finding of urinary or other organ |
| V1052, V4573 | Personal history of malignant neoplasm of the kidney, acquired absence of the kidney |
| **ICD-9 Procedure Codes** | **Description** |
| **Direct “Open” Radical Nephrectomy codes  (DORN)** |  |
| 5551 | Nephroureterectomy |
| 5552 | Nephrectomy of remaining kidney |
| 5554 | Bilateral nephrectomy |
| **Direct “Open” Partial Nephrectomy codes (DOPN)** |  |
| 554 | Partial nephrectomy |
| **Indirect “Open” Radical Nephrectomy codes (IORN)** |  |
| 3807 | Abdominal vein incision |
| **Indirect “Open” Partial Nephrectomy Codes (DOPN)** |  |
| 5501 | Nephrotomy |
| 5524 | Open biopsy of kidney |
| 5531 | Marsupialization of kidney lesion |
| 5539 | Other local excision/destruction of renal lesion |
| 5581 | Suture laceration of kidney |
| 5589 | Other repair of kidney |
| 5591 | Decapsulation of the kidney |
| 5902 | Other lysis perirenal/periureteral tissue |
| 5909 | Other incision perirenal/periureteral tissue |
| 5921 | Biopsy perirenal and perivesical tissues |
| **Indirect Laparoscopy Codes (LAP)** |  |
| 4711 | Incidental laparoscopic appendectomy |
| 5123 | Laparoscopic cholecystectomy |
| 5421 | Laparoscopy |
| 5451 | Laparoscopic lysis of peritoneal adhesions |
| 5903 | Laparoscopic lysis perirenal/periureteral adhesions |
| 6563 | Laparoscopic removal bilateral ovary-tubes |
| **CPT Procedure Codes** | **Description** |
| **Direct “Open” Radical Nephrectomy Codes (DORN)** |  |
| 50220 | Nephrectomy, including partial ureterectomy, any open approach |
| 50225 | Nephrectomy, including partial ureterectomy, any open approach, complicated |
| 50230 | Nephrectomy, radical, with regional lymphadenectomy and/or vena caval thrombectomy |
| 50234 | Nephrectomy, with total ureterectomy and bladder cuff through same incision |
| 50236 | Nephrectomy, with total ureterectomy and bladder cuff through separate incision |
| **Direct “Open” Partial Nephrectomy Codes (DOPN)** |  |
| 50240 | Nephrectomy, partial |
| **Direct “Laparoscopic” Radical Nephrectomy Codes (DLRN)** |  |
| 50545 | Laparoscopy, radical nephrectomy |
| 50546 | Laparoscopy, nephrectomy including partial ureterectomy |
| 50548 | Laparoscopy, nephrectomy with total ureterectomy |
| **Direct “Laparoscopic” Partial Nephrectomy Codes (DLPN)** |  |
| 50542 | Laparoscopy, surgical; ablation of renal mass lesion(s) |
| 50543 | Laparoscopy, partial nephrectomy |
| **Indirect “Open” Radical Nephrectomy Codes (IORN)** |  |
| 37799 | Unlisted procedure, vascular surgery (for vena caval resection with reconstruction) |
| **Indirect “Open” Partial Nephrectomy Codes (IOPN)** |  |
| 49010 | Exploration, retroperitoneal area with or without biopsy |
| 49200 | Excision or destruction, open, intra-abdominal or retroperitoneal tumors or cysts |
| 49201 | Excision or destruction, open, intra-abdominal or retroperitoneal tumors or cysts, extensive |
| 50205 | Renal biopsy by surgical exposure of the kidney |
| 50280 | Excision or unroofing of cysts(s) of kidney |
| 50380 | Renal autotransplantation, reimplantation of kidney |
| **Indirect “Laparoscopic” Radical Nephrectomy Codes (ILRN)** |  |
| 50549 | Unlisted laparoscopy procedure, renal |
| 56399 | Unlisted procedure, laparoscopy |
| 60650 | Laparoscopy with adrenalectomy |
| **Indirect “Laparoscopic” Partial Nephrectomy Codes (ILPN)** |  |
| 50541 | Laparoscopy, surgical; ablation of renal cysts |
| **Indirect Laparoscopy Codes (LAP)** |  |
| 44200 | Laparoscopic enterolysis |
| 47562 | Laparoscopic cholecystectomy |
| 49320 | Laparoscopy, diagnostic |
| 49321 | Laparoscopy diagnostic, with biopsy |
| 56300 | Laparoscopy, diagnostic |
| 56340 | Laparoscopic cholecystectomy |
