# Supplementary material for: External validation of a claims-based algorithm for classifying kidney-cancer surgeries
Source: BMC Health Serv Res. 2009 Jun 6;9:92. doi: 10.1186/1472-6963-9-92 (PMC2698842; doi:10.1186/1472-6963-9-92)
Supplement: Additional file 3 — Description of algorithms for surgical procedure assignment. The series of tables in Additional File 3 illustrate our algorithm for surgical procedure assignment. This algorithm included methods to resolve procedure assignment for cases with more than one relevant physician procedural claim (Additional File 3, Table A), more than one relevant inpatient (i.e., hospital) procedural claim (Additional File 3, Table B), and/or a discrepancy between inpatient and physician claims (Additional File 3, Table C). [file 1472-6963-9-92-S3.doc]

**Additional File 3. Description of algorithms for surgical procedure assignment**

The series of tables in Additional File 3 illustrate our algorithm for surgical procedure assignment. This algorithm included methods to resolve procedure assignment for cases with more than one relevant physician procedural claim (Additional File 3, Table A), more than one relevant inpatient (i.e., hospital) procedural claim (Additional File 3, Table B), and/or a discrepancy between inpatient and physician claims (Additional File 3, Table C). Definitions for the abbreviations presented in Additional File 3, Tables A, B, and C are provided in the Table for Additional File 2.

Because specific CPT codes for laparoscopic radical (introduced in 2000) and partial (introduced 2002) nephrectomy did not exist during the earlier years of the study, we estimated the proportion of laparoscopic cases based on an algorithm that incorporates both direct (CPT) and indirect (ICD-9 and CPT) laparoscopy codes. For instance, directly-identified laparoscopy cases included patients with a physician claim for laparoscopic radical nephrectomy (e.g., CPT code 50545) and a corresponding inpatient claim for nephrectomy (e.g., ICD-9 procedure code 5551). We assigned cases with this combination of codes to the laparoscopic nephrectomy cohort. An example of an indirectly identified laparoscopic radical nephrectomy is a patient with a physician claim for radical nephrectomy (e.g., CPT code 50230) and a corresponding inpatient claim with ICD-9 procedure codes for nephrectomy (e.g., 5551) and laparoscopic exploration (e.g., 5421).

Finally, after assigning each case to a procedure based on physician and inpatient claims data, we ascribed a laparoscopic approach to cases with a live discharge and length of stay ≤ 2 days following radical or partial nephrectomy. Based on the methodology described in this Additional File, we assigned each patient to one of four mutually exclusive surgical procedures: 1) open radical nephrectomy (ORN); 2) open partial nephrectomy (OPN); 3) laparoscopic radical nephrectomy (LRN); 4) laparoscopic partial nephrectomy (LPN).

**Additional File 3,Table A. Algorithm for reconciling physician claims (NCH file) with more than one kidney-cancer surgical procedure code. For abbreviation definitions, see Additional File 2.**

Table A presents our algorithm for surgical procedure assignment based on physician claims data. In this table, distinct categories of physician procedural claims are arranged along the first (top) row and first (left column). ***For cases with more than one physician claim (including discrepant claims), we specify the number of cases and the final procedure assignment (from physician claims) in the cell that corresponds with the row and column for the two separate claims.***

|  | **DORN** | **DOPN** | **DLRN** | **DLPN** | **IORN** | **IOPN** | **ILRN** | **ILPN** | **LAP** |
| --- | --- | --- | --- | --- | --- | --- | --- | --- | --- |
| **DORN** | DORN | DOPN  (*n*=23) | LRN  (*n*=15) | LPN | DORN  (*n*=15) | DORN  (*n*=24) | LRN | LRN  (*n*=1) | LRN  (*n*=18) |
| **DOPN** | DOPN | DOPN | LRN  (*n*=1) | LPN | DOPN  (*n*=1) | DOPN  (*n*=16) | LPN | LPN  (*n*=3) | LPN  (*n*=1) |
| **DLRN** | LRN | LPN | LRN | LPN | LRN  (*n*=3) | LRN  (*n*=1) | LRN | LRN | LRN  (*n*=1) |
| **DLPN** | LPN | LPN | LPN | LPN | LPN | LPN  (*n*=1) | LPN | LPN | LPN |
| **IORN** | DORN | DOPN | LRN | LPN | IORN | IOPN | LRN | LRN | LRN |
| **IOPN** | DORN | DOPN | LRN | LPN | IOPN | IOPN | LPN  (*n*=2) | LPN  (*n*=2) | LPN |
| **ILRN** | LRN | LPN | LPN | LRN | LPN | LRN | LRN | LPN  (*n*=2) | LRN |
| **ILPN** | LRN | LPN | LRN | LPN | LRN | LPN | LPN | LPN | LPN  (*n*=1) |
| **LAP** | LRN | LPN | LRN | LPN | LRN | LPN | LRN | LPN | LAP ONLY |

**Additional File 3,Table B. Algorithm for reconciling hospital claims (NCH file) specifying more than one kidney-cancer surgical procedure code. For abbreviation definitions, see Additional File 2.**

Table B presents our algorithm for surgical procedure assignment based on inpatient (i.e., hospital) claims data. In this table, distinct categories of inpatient procedural claims are arranged along the first (top) row and first (left column). ***For cases with more than one relevant inpatient claim (including discrepant claims), we specify the number of cases and the final procedure assignment (from inpatient claims) in the cell that corresponds with the row and column for the two separate claims.***

|  | **DORN** | **DOPN** | **IORN** | **IOPN** | **LAP** |
| --- | --- | --- | --- | --- | --- |
| **DORN** | DORN | DOPN  (*n*=10) | DORN  (*n*=98) | DORN  (*n*=36) | LRN  (*n*=202) |
| **DOPN** | DOPN | DOPN | DOPN | DOPN  (*n*=41) | LPN  (*n*=12) |
| **IORN** | DORN | DOPN | IORN | IOPN | LRN |
| **IOPN** | DORN | DOPN | IOPN | IOPN | LPN  (*n*=7) |
| **LAP** | LRN | LPN | LRN | LPN | LAP ONLY |

**Additional File 3, Table C. Algorithm for reconciling discrepancies between physician and inpatient claims (number of cases in the final analytic cohort falling within a particular cell) *. For abbreviation definitions, see Additional File 2.**

**Table C presents our algorithm for the final step in surgical procedure assignment. Specifically, this table describes our method for combining physician and inpatient procedural claims for the same case. In this table, distinct categories of physician procedural claims (based on CPT codes) are arranged along first (left) column; distinct categories of inpatient procedural claims (based on ICD-9 procedural codes) are arranged along the first (top) row. *For cases with both a physician and inpatient procedural claim (including discrepant claims), we specify the number of cases and the final procedure assignment in the cell that corresponds with the row and column for the two separate claims.***

|  | **PROCEDURE BASED ON INPATIENT CLAIMS (ICD-9 codes)** | | | | | | | |
| --- | --- | --- | --- | --- | --- | --- | --- | --- |
|  |  | **DORN** | **DOPN** | **LRN** | **LPN** | **IORN** | **IOPN** | **LAP ONLY** |
| **PROCEDURE BASED ON PHYSICIAN CLAIMS (CPT codes)** | **DORN** | ORN (*n*=4,012) | OPN (*n*=40) | LRN (*n*=50) | LPN (*n*=1) | ORN | ORN (*n*=6) | LRN (*n*=1) |
|  | **DOPN** | OPN (*n*=42) | OPN (*n*=429) | LPN (*n*=1) | LPN (*n*=9) | OPN | OPN (*n*=6) | LPN |
|  | **LRN** | LRN (*n*=232) | LRN (*n*=16) | LRN (*n*=142) | LPN (*n*=5) | LRN | LRN (*n*=4) | LRN |
|  | **LPN** | LPN | LPN (*n*=5) | LPN | LPN (*n*=5) | LPN | LPN (*n*=6) | LPN |
|  | **IORN** | ORN (*n*=1) | OPN | LRN | LPN | LRN | ORN | LRN |
|  | **IOPN** | ORN (*n*=1) | OPN (*n*=1) | LPN | LPN | LPN | OPN (*n*=13) | LPN |
|  | **LAP ONLY** | LRN | LPN | LRN | LPN | LRN | LPN | LAP ONLY (EXCLUDE) |

* The final procedure assignment for cases that had only a physician or an inpatient claim is as follows: (1) We assigned 2 cases with physician claims for CPT-DOPN and no inpatient procedural claim to OPN; (2) We assigned 14 cases with physician claims for CPT-DORN and no inpatient procedural claim to ORN; (3) We assigned 2 cases with physician claims for CPT-IOPN and no inpatient procedural claim to OPN; (4) We assigned 1 case with a physician claim for CPT-LPN and no inpatient procedural claim to LPN; (5) We assigned 39 cases with inpatient procedural claims for ICD-DOPN and no physician procedural claim to OPN; (6) We assigned 379 cases with inpatient procedural claims for ICD-DORN and no physician procedural claim to ORN; (7) We assigned 9 cases with inpatient procedural claims for ICD-IOPN and no physician procedural claim to OPN; (8) We assigned 9 cases with inpatient procedural claims for ICD-LRN and no physician procedural claim to LRN; (9) We excluded from analysis 9 cases with inpatient claims for ICD-LAP ONLY and no physician procedural claims.
