# Supplementary material for: External validation of a claims-based algorithm for classifying kidney-cancer surgeries
Source: BMC Health Serv Res. 2009 Jun 6;9:92. doi: 10.1186/1472-6963-9-92 (PMC2698842; doi:10.1186/1472-6963-9-92)
Supplement: Additional file 4 — Distribution of patient characteristics. The series of tables in Additional File 4 summarize the distribution of patient characteristics for each of the patient cohorts and sub-cohorts specified in the manuscript. [file 1472-6963-9-92-S4.doc]

**Additional File 4, Table A. Distribution of patient characteristics for preliminary cohort (n=6,515)**

|  | ***n* (%)** |
| --- | --- |
| **Total** | 6,515 |
|  |  |
| **Patient-level covariates** |  |
| **Age** |  |
| *66–69 years* | 1,465 (22.5) |
| *70–74 years* | 1,999 (30.7) |
| *75–79 years* | 1,671 (25.6) |
| *80–84 years* | 965 (14.8) |
| *≥ 85 years* | 415 (6.4) |
| **Sex** |  |
| *Male* | 3,892 (59.7) |
| *Female* | 2,623 (40.3) |
| **Race/ ethnicity** |  |
| *White, Non-Hispanic* | 5,369 (82.4) |
| *White, Hispanic* | 391 (6.0) |
| *Black* | 532 (8.2) |
| *Other or unknown race/ethnicity* | 223 (3.4) |
| **Marital Status ^†^** |  |
| *Married* | 3,884 (62.0) |
| *Not married* | 2,380 (38.0) |
| **SEER Registry** |  |
| *Atlanta* | 203 (3.1) |
| *Connecticut* | 571 (8.8) |
| *Detroit* | 798 (12.3) |
| *Greater California* | 711 (10.9) |
| *Hawaii* | 80 (1.2) |
| *Iowa* | 629 (9.7) |
| *Kentucky* | 438 (6.7) |
| *Los Angeles* | 653 (10.0) |
| *Louisiana* | 379 (5.8) |
| *New Jersey* | 851 (13.1) |
| *New Mexico* | 203 (3.1) |
| *Rural Georgia* | 21 (0.3) |
| *San Francisco* | 236 (3.6) |
| *San Jose* | 156 (2.4) |
| *Seattle* | 412 (6.3) |
| *Utah* | 174 (2.7) |
| **Median census tract income** ‡ |  |
| *< $ 35,000* | 2,670 (41.2) |
| *$ 35,000–$ 44,999* | 1,531 (23.6) |
| *$ 45,000–$ 59,999* | 1,303 (20.1) |
| *≥ $ 60,000* | 974 (15.1) |
| **Percentage of residents in Census tract with less than high school education ^§^** |  |
| *> 25.0* | 2,041 (31.5) |
| *15.1–25.0* | 1,928 (29.8) |
| *10.0 –15.0* | 1,068 (16.5) |
| *< 10.0* | 1,441 (22.2) |
| **Tumor Size ^^** |  |
| *≤ 4 cm* | 2,766 (44.5) |
| *> 4 cm* | 3,450 (55.5) |

**^†^** marital status unknown for 251 cases; ‡ income missing for 37 cases; **^§^** education missing for 37 cases; **^^** tumor size missing for 299 cases.

Additional File 4, Table B. Distribution of patient characteristics for analytic cohort (n=5,483)

|  | ***n* (%)** |
| --- | --- |
| **Total** | 5,483 |
|  |  |
| **Patient-level covariates** |  |
| **Age** |  |
| *66–69 years* | 1,285 (23.4) |
| *70–74 years* | 1,755 (32.0) |
| *75–79 years* | 1,407 (25.6) |
| *80–84 years* | 779 (14.2) |
| *≥ 85 years* | 257 (4.7) |
| **Sex** |  |
| *Male* | 3,219 (58.7) |
| *Female* | 2,264 (41.3) |
| **Race/ ethnicity** |  |
| *White, Non-Hispanic* | 4,563 (83.2) |
| *White, Hispanic* | 335 (6.1) |
| *Black* | 396 (7.2) |
| *Other or unknown race/ethnicity* | 189 (3.5) |
| **Marital Status ^†^** |  |
| *Married* | 3,353 (63.6) |
| *Not married* | 1,919 (36.4) |
| **SEER Registry** |  |
| *Atlanta* | 170 (3.1) |
| *Connecticut* | 501 (9.1) |
| *Detroit* | 667 (12.2) |
| *Greater California* | 591 (10.8) |
| *Hawaii* | 64 (1.2) |
| *Iowa* | 534 (9.7) |
| *Kentucky* | 364 (6.6) |
| *Los Angeles* | 549 (10.0) |
| *Louisiana* | 322 (5.9) |
| *New Jersey* | 725 (13.2) |
| *New Mexico* | 168 (3.1) |
| *Rural Georgia* | 17 (0.3) |
| *San Francisco* | 195 (3.6) |
| *San Jose* | 137 (2.5) |
| *Seattle* | 334 (6.1) |
| *Utah* | 145 (2.6) |
| **Median census tract income** ‡ |  |
| *< $ 35,000* | 2,182 (40.0) |
| *$ 35,000–$ 44,999* | 1,310 (24.0) |
| *$ 45,000–$ 59,999* | 1,115 (20.5) |
| *≥ $ 60,000* | 843 (15.5) |
| **Percentage of residents in Census tract with less than high school education ^§^** |  |
| *> 25.0* | 1,670 (30.6) |
| *15.1–25.0* | 1,635 (30.0) |
| *10.0 –15.0* | 911 (16.7) |
| *< 10.0* | 1,234 (22.6) |
| **Tumor Size ^^** |  |
| *≤ 4 cm* | 2,340 (44.2) |
| *> 4 cm* | 2,960 (55.8) |

**^†^** marital status unknown for 211 cases; ‡ income missing for 33 cases; **^§^** education missing for 33 cases; **^^** tumor size missing for 183 cases.

**Additional File 4, Table C**. Distribution of patient characteristics for LA cases in analytic cohort (n=549)

|  | ***n* (%)** |
| --- | --- |
| **Total** | 549 |
|  |  |
| **Patient-level covariates** |  |
| **Age** |  |
| *66–69 years* | 136 (24.8) |
| *70–74 years* | 156 (28.4) |
| *75–79 years* | 132 (24.0) |
| *80–84 years* | 94 (17.1) |
| *≥ 85 years* | 31 (5.7) |
| **Sex** |  |
| *Male* | 331 (60.3) |
| *Female* | 218 (39.7) |
| **Race/ ethnicity** |  |
| *White, Non-Hispanic* | 385 (70.1) |
| *White, Hispanic* | 75 (13.7) |
| *Black* | 48 (8.7) |
| *Other or unknown race/ethnicity* | 41 (7.5) |
| **Marital Status ^†^** |  |
| *Married* | 327 (60.4) |
| *Not married* | 214 (39.6) |
| **Median census tract income** |  |
| *< $ 35,000* | 175 (31.9) |
| *$ 35,000–$ 44,999* | 120 (21.8) |
| *$ 45,000–$ 59,999* | 129 (23.5) |
| *≥ $ 60,000* | 125 (22.8) |
| **Percentage of residents in Census tract with less than high school education** |  |
| *> 25.0* | 180 (32.8) |
| *15.1–25.0* | 102 (18.6) |
| *10.0 –15.0* | 96 (17.5) |
| *< 10.0* | 171 (31.2) |
| **Tumor Size ^^** |  |
| *≤ 4 cm* | 238 (44.3) |
| *> 4 cm* | 299 (55.7) |

**^†^** marital status unknown for 8 cases; **^^** tumor size missing for 12 cases.

**Additional File 4, Table D. Distribution of patient characteristics for sub-**cohort of LA Cases, including cases with medical record review (n=120) and cases for whom medical records could not be identified (n=21) (total n=141)

|  | ***n* (%)** |
| --- | --- |
| **Total** | 141 |
|  |  |
| **Patient-level covariates** |  |
| **Age** |  |
| *66–69 years* | 36 (25.5) |
| *70–74 years* | 40 (28.4) |
| *75–79 years* | 38 (26.9) |
| *80–84 years* | 19 (13.5) |
| *≥ 85 years* | 8 (5.7) |
| **Sex** |  |
| *Male* | 89 (63.1) |
| *Female* | 52 (36.9) |
| **Race/ ethnicity** |  |
| *White, Non-Hispanic* | 95 (67.4) |
| *White, Hispanic* | 21 (14.9) |
| *Black* | 10 (7.1) |
| *Other or unknown race/ethnicity* | 15 (10.6) |
| **Marital Status** |  |
| *Married* | 85 (60.3) |
| *Not married* | 56 (39.7) |
| **Median census tract income** |  |
| *< $ 35,000* | 45 (31.9) |
| *$ 35,000–$ 44,999* | 24 (17.0) |
| *$ 45,000–$ 59,999* | 40 (28.4) |
| *≥ $ 60,000* | 32 (22.7) |
| **Percentage of residents in Census tract with less than high school education** |  |
| *> 25.0* | 47 (33.3) |
| *15.1–25.0* | 30 (21.3) |
| *10.0 –15.0* | 21 (14.9) |
| *< 10.0* | 43 (30.5) |
| **Tumor Size ^^** |  |
| *≤ 4 cm* | 61 (43.9) |
| *> 4 cm* | 78 (56.1) |

**^^** tumor size missing for 2 cases.

Additional File 4, Table E. Distribution of patient characteristics for medical record validation sample (n=120)

|  | ***n* (%)** |
| --- | --- |
| **Total** | 120 |
|  |  |
| **Patient-level covariates** |  |
| **Age** |  |
| *66–69 years* | 29 (24.1) |
| *70–74 years* | 35 (29.2) |
| *75–79 years* | 32 (26.7) |
| *80–84 years* | 16 (13.3) |
| *≥ 85 years* | 8 (6.7) |
| **Sex** |  |
| *Male* | 73 (60.8) |
| *Female* | 47 (39.2) |
| **Race/ ethnicity** |  |
| *White, Non-Hispanic* | 86 (71.7) |
| *White, Hispanic* | 16 (13.3) |
| *Black* | 7 (5.8) |
| *Other or unknown race/ethnicity* | 11 (9.2) |
| **Marital Status** |  |
| *Married* | 71 (59.2) |
| *Not married* | 49 (40.8) |
| **Median census tract income** |  |
| *< $ 35,000* | 34 (28.3) |
| *$ 35,000–$ 44,999* | 21 (17.5) |
| *$ 45,000–$ 59,999* | 35 (29.2) |
| *≥ $ 60,000* | 30 (25.0) |
| **Percentage of residents in Census tract with less than high school education** |  |
| *> 25.0* | 35 (29.2) |
| *15.1–25.0* | 25 (20.8) |
| *10.0 –15.0* | 21 (17.5) |
| *< 10.0* | 39 (32.5) |
| **Tumor Size ^^** |  |
| *≤ 4 cm* | 56 (47.5) |
| *> 4 cm* | 62 (52.5) |

**^^** tumor size missing for 2 cases.
